# Designing 2D–2D g-C_3_N_4_/Ag:ZnIn_2_S_4_ nanocomposites for the high-performance conversion of sunlight energy into hydrogen fuel and the meaningful reduction of pollution†

**DOI:** 10.1039/d0ra06226j

**Published:** 2020-09-03

**Authors:** Yu Gao, Kun Qian, Baotong Xu, Fu Ding, Valerian Dragutan, Ileana Dragutan, Yaguang Sun, Zhenhe Xu

**Affiliations:** The Key Laboratory of Inorganic Molecule-Based Chemistry of Liaoning Province, College of Materials and Engineering, Shenyang University of Chemical Technology Shenyang 110142 P. R. China dingfu@syuct.edu.cn xuzh@syuct.edu.cn; Key Laboratory of Resource Chemical Technology and Materials (Ministry of Education), Shenyang University of Chemical Technology Shenyang 110142 PR China; Institute of Organic Chemistry, Romanian Academy Spl. Independentei 202B 060023 Bucharest Romania idragutan@yahoo.com

## Abstract

The generation of hydrogen-based energy and environmental remediation using sunlight is an emerging topic of great significance for meeting the ever-growing global need. However, the actual photocatalytic performance is still far below expectations because of the relatively slack charge-carrier separation and migration as well as insufficient spectral absorption in semiconductors. Therefore, the rational construction of heterojunctions is considered as an effective approach to solving the above issues. In this context, we have, for the first time, designed and synthesized a two-dimensional 2D-on-2D heterostructure, based on 2D Ag-doped ZnIn_2_S_4_ nanoplates deposited on 2D g-C_3_N_4_ nanosheets (denoted as g-C_3_N_4_/Ag:ZnIn_2_S_4_). This construct benefits from improved visible-light absorption by unveiling a greater number of catalytically active sites, effectively enhancing charge-carrier separation and relocation. Detailed analysis has proved that under visible-light irradiation, the optimized g-C_3_N_4_/20 wt% Ag:ZnIn_2_S_4_ nanocomposite has substantially upgraded photocatalytic activity in hydrogen formation by water splitting (hydrogen evolution rate of up to 597.47 μmol h^−1^ g^−1^) and in residual dyestuff degradation (methyl orange, MO; degradation rate constant of 0.1406 min^−1^). Noteworthily, these two exceptionally high values respectively represent 30.73 and 5.42 times enhancements *vs.* results obtained with bare g-C_3_N_4_. Another strong point of our g-C_3_N_4_/Ag:ZnIn_2_S_4_ is its impressive recyclability for 20 runs, with no relevant metal release in the aqueous solution following photocatalysis. This work introduces new, superior access to highly efficient photocatalysts founded on 2D/2D nanocomposites serving both the production of hydrogen as an energy carrier and environmental remediation.

## Abstract 2

The generation of hydrogen-based energy and environmental remediation using sunlight is an emerging topic of great significance for meeting the ever-growing global need.

## Graphical abstract

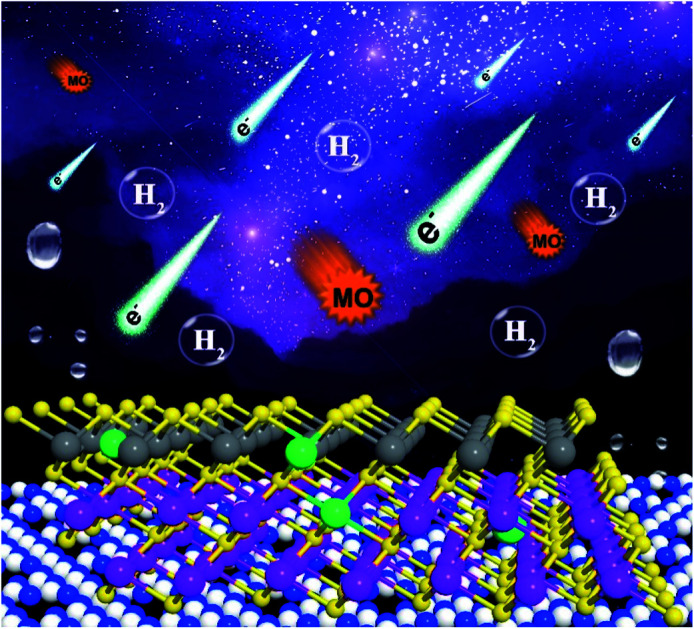

## 1 Introduction

Sunlight, as an energy supply, is widely available and costless and so is water. Valorizing the energy in sunlight to produce hydrogen from water has long been considered as advantageous, more so that this much-coveted fuel is acquired under photocatalysis without other energy conversions. In the last decades, the search for sustainable photocatalysis-based technologies and the required materials became an arduous pursuit that can additionally mitigate some crucial issues related to the planetary energy shortage and environmental damage.^1–9^

Following the first report by Fujishima and Honda on the photocatalytic assets of TiO_2_,^10^ an impressive number of strategies have been devised to create quite efficient, stable, inexpensive and broadband-responsive photocatalysts.^11–14^ Among them, graphitic carbon nitride (g-C_3_N_4_) stands out after Wang *et al.* had proved it, in 2009, as a promising visible-light responsive photocatalyst.^15^ Two-dimensional (2D) g-C_3_N_4_ can offer an extended specific surface area, reveal supplemental exposed active sites, provide an easier approach and diffusion paths for both ions and electrons.^16–18^ However, a single component cannot satisfy all requirements because of the low efficiency of solar energy conversion, the less-than-ideal solar light response range, the low ability for charge separation/transfer, and reduced photocatalytic stability.^19,20^ To solve the aforementioned problems, some typical protocols have been conceived to enhance the photocatalytic process.^21–23^ One of the most effective tactics is the construction of multicomponent/multiphase heterostructured photocatalysts comprised of distinct units that arise from physical–chemical interactions such as covalent or ionic bonding, hydrogen bonding, van der Waals (VDW) and Coulomb forces.^1,24–28^ Furthermore, devising 2D/2D heterojunctions is a most adequate approach towards an enhanced photocatalytic achievement. Owing to their “face to face” contact, such heterojunctions could ensure improved interfacial charge separation and transfer between layers, also enabling broad light-responsivity with different bandgap widths of the two photocatalytic semiconductors.^29^ Unfortunately, the photocatalytic performance of 2D/2D heterojunctions is presently still disappointing. The design of g-C_3_N_4_-based 2D/2D heterojunctions from selected components with matchable bandgaps, able to harvest solar light efficiently and economically is, therefore, in high demand.

Recently, Ag^+^-doped ZnIn_2_S_4_ with a 2D multi-layered structure has shown superiority in photocatalytic hydrogen evolution mainly because of suitable bandgap management of the composition-dependent absorbed light.^19,20^ The silver ions can create acceptor and donor states through substitutional doping and interstitial doping, which significantly improve the carrier density and charge transport efficiency and accordingly increase the photocatalytic activity.^30^ More importantly, 2D Ag:ZnIn_2_S_4_ nanosheets can be prepared using a straightforward hydrothermal procedure to construct 2D/2D heterojunctions. As far as we know, there are no reports on a 2D/2D g-C_3_N_4_/Ag:ZnIn_2_S_4_ heterojunction applied as a photocatalyst. Furthermore, the bandgap of Ag:ZnIn_2_S_4_ (*E*_g_ = ∼2.61 eV) is smaller than that of g-C_3_N_4_ (*E*_g_ = ∼2.76 eV), while the position of the conduction band (CB) of Ag:ZnIn_2_S_4_ (*E*_CB_Ag:ZnIn_2_S_4_ = −1.47 eV) has a higher negative value than that of g-C_3_N_4_ (*E*_CB_C_3_N_4_ = −1.18 eV) and thus provides a better opportunity for the directional transfer of the electrons photogenerated from Ag:ZnIn_2_S_4_ towards g-C_3_N_4_. Moreover, due to the position of the valence band (VB) of g-C_3_N_4_ (*E*_VB_C_3_N_4_ = +1.58 eV) having a higher positive value than that of Ag:ZnIn_2_S_4_ (*E*_VB_Ag:ZnIn_2_S_4_ = +1.14 eV), the holes on the VB of g-C_3_N_4_ will migrate to that of Ag:ZnIn_2_S_4_, achieving the separation of the charge carriers in heterojunctions. It can thus be anticipated that by integrating g-C_3_N_4_ with Ag:ZnIn_2_S_4_ a “type II” heterojunction will be formed at their interface, which may result in improved photocatalytic activity.

Stimulated by the above-mentioned considerations we, for the first time, report here the assembly of a 2D/2D g-C_3_N_4_/Ag:ZnIn_2_S_4_ heterojunction by the *in situ* growth of ultrathin Ag:ZnIn_2_S_4_ nanosheets on g-C_3_N_4_ nanosheets using a hydrothermal protocol. This new idea is valorized for the enhanced visible-light-driven H_2_ evolution from water and for the photodegradation of the dye methyl orange (MO) contained in waste industrial effluents. Our work can serve as an inspiration in the ongoing global research effort for developing materials based on “face to face” 2D/2D heterojunctions destined for the photocatalytic generation of hydrogen as an energy carrier.

## 2 Experimental section

### 2.1 Materials

Thiourea, silver nitrate (AgNO_3_), zinc acetate (Zn(OAc)_2_·2H_2_O), indium acetate (In(OAc)_3_), l-cysteine (l-cys), thioacetamide (TAA), methyl orange (MO), 1,4-benzoquinone (BQ), disodium ethylenediaminetetraacetate (Na_2_EDTA), and *tert*-butyl alcohol (*t*-BuOH) were purchased from Aladdin Reagent Co. Ltd. All chemicals were analytically pure and were used without further purification. Pure water, purified by a Millipore ultrapure water system and having a resistivity of 18.2 MΩ cm (at 25 °C), was used in the current investigation.

### 2.2 Synthesis of the g-C_3_N_4_/Ag:ZnIn_2_S_4_ heterojunctions

First, g-C_3_N_4_ was obtained by a traditional thermal polymerization method.^2,31^ Secondly, the g-C_3_N_4_/Ag:ZnIn_2_S_4_ heterojunctions were synthesized by the *in situ* growth of ultrathin Ag:ZnIn_2_S_4_ nanosheets on g-C_3_N_4_ nanosheets through a facile hydrothermal method. In a typical procedure, a specific amount of g-C_3_N_4_ was ground into fine powder and then added to 20 mL of distilled water. After ultrasonic treatment for 6 h, the g-C_3_N_4_ was exfoliated into thin nanosheets which were then collected and washed using centrifugation–redispersion with deionized water. Subsequently, the exfoliated g-C_3_N_4_ nanosheets were resuspended into 50 mL of a premade aqueous solution consisting of the calculated amount of Zn(OAc)_2_·2H_2_O, In(OAc)_3_, AgNO_3_ and l-cys. After being stirred for 30 min, TAA was added to form a homogeneous solution. This solution was transferred into a 100 mL Teflon-lined autoclave and maintained at 160 °C for 6 hours. After cooling the autoclave to room temperature, the solid was collected, washed several times with ethanol and deionized water, and finally dried overnight in an electric oven at 60 °C. According to this method, samples with different weight ratios of g-C_3_N_4_/Ag:ZnIn_2_S_4_, *i.e.*, having Ag:ZnIn_2_S_4_ contents of 10 wt%, 20 wt% and 30 wt%, were synthesized. The actual contents of Ag:ZnIn_2_S_4_ in the composites were measured by inductively coupled plasma-optical emission spectrometry (ICP-OES). The measured values, 10.4, 19.6 and 30.7 wt%, were quite close to the nominal loading mass fractions, 10, 20, and 30 wt%. Pure Ag:ZnIn_2_S_4_ was similarly prepared without introducing g-C_3_N_4_.

### 2.3 Characterization

The X-ray diffraction (XRD) patterns of the samples were collected on a Bruker D8 Advance X-ray diffractometer with Ni-filtered Cu Kα radiation, at 40 kV and 40 mA, with 2*θ* ranging from 10° to 80° and with a scan rate of 0.02° per second. Transmission electron microscopy (TEM) images were obtained using a JEOL model JEM 2010 EX instrument at an accelerating voltage of 200 kV. UV-vis diffused reflectance spectra of the samples were obtained from a UV-vis spectrophotometer (UV2550, Shimadzu, Japan), with BaSO_4_ being used as a reflectance standard. The photoluminescence (PL) spectra were measured by using a fluorescence spectrophotometer (F4500, Hitachi, Japan) with the excitation wavelength of 260 nm. Inductively coupled plasma optical emission spectroscopy (ICP-OES; Shimadzu Co., ICPS-8100) was used to quantify the elements present. X-ray photoelectron spectroscopy (XPS) measurements were performed in a Thermal ESCALAB 250Xi electron spectrometer using Al Kα radiation X-ray source (*hν* = 1486.6 eV) to determine the chemical states and the valence states of the involved elements. The photoelectrochemical (PEC) measurements were performed with an electrochemical workstation (CHI 660E, CH Instruments) in a standard three-electrode cell, using a Pt wire and a Ag/AgCl electrode (3 M KCl) as the counter and reference electrode, respectively. The working electrode was prepared on fluorine-doped tin oxide (FTO) glass with its boundary being protected by Scotch tape. Five milligrams of as-synthesized powder were dispersed in 1 mL of dimethylformamide under sonication for 30 min to obtain a colloidal dispersion. The dispersion was drop-cast onto the FTO glass. After natural air-drying, the uncoated part of the FTO glass was isolated with epoxy resin glue. Then, 0.2 M of Na_2_SO_4_ (pH = 6.8) aqueous solution was pre-purged with nitrogen for 30 min and was used as an electrolyte. Nyquist plots were recorded over the frequency range of 100 mHz to 100 kHz at a bias of 0.2 V.

### 2.4 Photocatalytic hydrogen evolution from water

The photocatalytic reaction was performed in the Perfect Light Labsolar-6A automatic online photocatalytic analysis system (Labsolar-6A, Beijing Perfect light Co. Ltd.). The reaction was carried out by mixing an aqueous solution (100 mL) of the photocatalyst (50 mg) with 10 vol% triethanolamine solution as a sacrificial agent for hydrogen production. Before starting the photocatalytic reaction, the solution was purged with Ar gas for 30 min. A 300 W xenon lamp (PLS-SXE 300, Beijing Perfect light Co. Ltd.) with a UV-Cut filter to cut off the light of wavelength *λ* < 420 nm was used as the irradiation source. During testing, the solution was continuously stirred with a magnetic stirrer while the reaction solution was maintained at room temperature by a water-cooling system. A Shimadzu GC-2014 gas chromatograph, equipped with a thermal conductivity detector and high-purity Ar gas carrier, was used to analyze the reaction-evolved gases. Subsequently, the sample resulting from the first reaction run was again subjected to the photocatalytic H_2_ evolution experiment, under identical conditions; the procedure was repeated for 20 cycles with a total irradiation time of 100 h.

### 2.5 Photodegradation of MO and detection of active species

The photodegradation activity of the as-prepared g-C_3_N_4_/Ag:ZnIn_2_S_4_ photocatalysts was evaluated for the removal of MO from contaminated water under visible light irradiation. An amount of 10 mg of the g-C_3_N_4_/Ag:ZnIn_2_S_4_ photocatalyst was added to 20 mL of MO solution (10 mg L^−1^), in a 100 mL quartz reactor equipped with circulating cooling water to keep the reaction temperature constant. Prior to irradiation, the suspension was stirred in the dark for 30 min to reach adsorption–desorption equilibrium between the photocatalyst and MO. A 300 W xenon lamp (PLS-SXE 300, Beijing Perfect light Co. Ltd.) filtered by a UV cut-off filter (*λ* > 420 nm) was used as the visible light source. The solution was irradiated with the above-mentioned light under magnetic stirring. At different irradiation times, 0.5 mL aliquots were taken out and centrifuged to separate the MO solution from the photocatalyst. The MO residue in the supernatant was quantified using UV-vis spectrometry (Shimadzu UV-2550), following the absorbance at 465 nm. Thus, 20 successive runs were carried out on the recovered samples, under identical conditions to test the MO degradation rate. Furthermore, in order to detect the active species involved in the photocatalytic removal of MO, trapping experiments were performed using different scavengers. Specifically, 1,4-benzoquinone (BQ) (1 mM), disodium ethylenediaminetetraacetate (Na_2_EDTA) (1 mM), and *tert*-butyl alcohol (*t*-BuOH) (1 mM) served as superoxide radical (˙O^2−^), hole, and hydroxyl radical (˙OH) scavengers, respectively.

## 3 Results and discussion

**Scheme 1:**
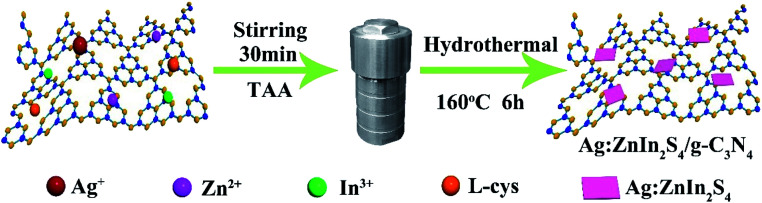
Schematic illustration of the fabrication process of g-C_3_N_4_/Ag:ZnIn_2_S_4_ nanocomposites.

**Fig. 1:**
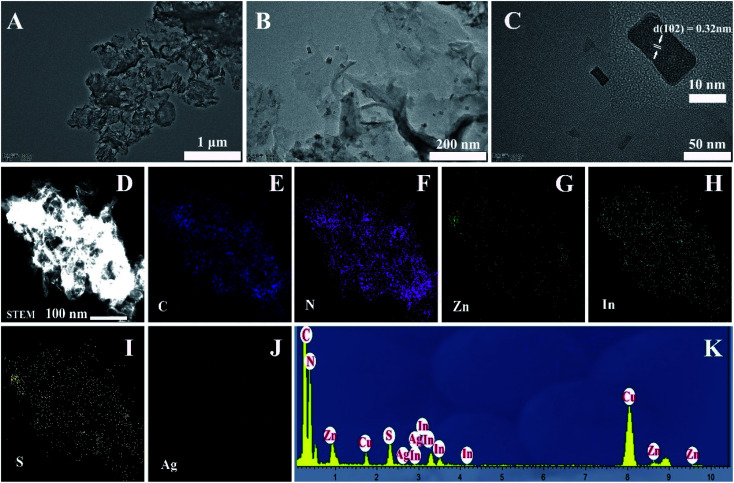
TEM images of (A) g-C_3_N_4_; (B and C) g-C_3_N_4_/20 wt% Ag:ZnIn_2_S_4_ photocatalyst with different magnifications. The inset in (C) is the HRTEM image of Ag:ZnIn_2_S_4_. (D–J) HAADF-STEM image and STEM-EDX mapping of C, N, Zn, In, S, and Ag elements. (K) EDS analysis.

A two-step synthesis route was employed to obtain the g-C_3_N_4_/Ag:ZnIn_2_S_4_ photocatalysts (Scheme 1): (i) synthesis of the 2D g-C_3_N_4_ nanosheet through thermal polymerization and subsequent ultrasonic exfoliation treatment (TEM image in Fig. 1A); (ii) hydrothermal deposition of Ag:ZnIn_2_S_4_ nanosheets onto the surface of g-C_3_N_4_ nanosheets. Subsequently, ultrathin Ag:ZnIn_2_S_4_ nanosheets were grown on the g-C_3_N_4_ nanosheets using a hydrothermal process. A representative TEM image (Fig. 1B) of this g-C_3_N_4_/Ag:ZnIn_2_S_4_ nanocomposite shows that a large number of Ag:ZnIn_2_S_4_ ultrathin nanosheets, of 10 nm in length and 5 nm in width, was observed on the g-C_3_N_4_ nanosheets surface. The Ag:ZnIn_2_S_4_ nanosheets were grown with face-to-face intimate contact on the g-C_3_N_4_ nanosheets acting as substrate (Fig. 1C). It is noteworthy is that no free Ag:ZnIn_2_S_4_ nanosheets were identified elsewhere, suggesting that a high loading efficiency was operative between the Ag:ZnIn_2_S_4_ and the g-C_3_N_4_ nanosheets. The high-resolution TEM image (Fig. 1C, inset) of an individual Ag:ZnIn_2_S_4_ ultrathin nanosheet deposited on the g-C_3_N_4_ surface provided evidence of crystal lattices with the interplanar distance of ≈0.32 nm, which is in accordance with the lattice spacing of the (102) facet of the ZnIn_2_S_4_. Note that a pure Ag:ZnIn_2_S_4_ sample, synthesized by the same hydrothermal process in the absence of g-C_3_N_4_ nanosheets, gave specific network structures; the individual structures could not be resolved (Fig. S1, ESI†). The high-angle annular dark-field scanning transmission electron microscopy (HAADF-STEM) image (Fig. 1D), combined with elemental mapping (Fig. 1E–J) and EDS analysis (Fig. 1K) confirmed the homogeneous distribution of the C, N, Zn, In, S and Ag elements in the nanocomposite.

**Fig. 2:**
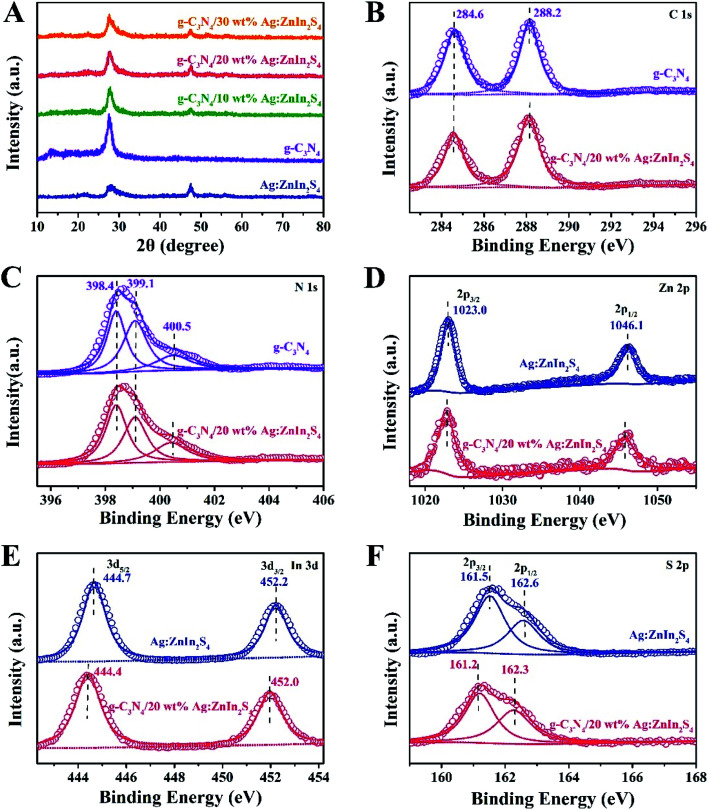
(A) XRD patterns of g-C_3_N_4_, Ag:ZnIn_2_S_4_, and g-C_3_N_4_/Ag:ZnIn_2_S_4_ samples. High-resolution (B) C 1s and (C) N 1s XPS spectra of g-C_3_N_4_ and g-C_3_N_4_/20 wt% Ag:ZnIn_2_S_4_ samples, (D) Zn 2p, (E) In 3d, and (F) S 2p XPS spectra of Ag:ZnIn_2_S_4_ and g-C_3_N_4_/20 wt% Ag:ZnIn_2_S_4_ samples.

X-ray diffraction (XRD) served to characterize the phase composition and crystal structure of the as-prepared g-C_3_N_4_, Ag:ZnIn_2_S_4_, and g-C_3_N_4_/Ag:ZnIn_2_S_4_ samples (Fig. 2A). In the XRD pattern of the g-C_3_N_4_ nanosheets, there are two pronounced diffraction peaks with 2*θ* = 13.2° and 27.5°, arising from the in-plane intervals of the periodic tri-*s*-triazine units as the (100) plane, and the specific interplanar stacking of aromatic systems as the (002) plane of graphite-like materials, respectively. The characteristic XRD peaks of Ag:ZnIn_2_S_4_ were well indexed to the hexagonal *P*6_3_*mc* space group of ZnIn_2_S_4_ (JCPDS card no. 72-0773). We did not observe any unidentifiable diffraction peaks, indicating that the Ag^+^ ions were incorporated into the lattice of ZnIn_2_S_4_, and did not form a separate phase. As expected, the as-prepared g-C_3_N_4_/Ag:ZnIn_2_S_4_ nanocomposites displayed peaks characteristic of both g-C_3_N_4_ and Ag:ZnIn_2_S_4_. Furthermore, by simply adjusting the hydrothermal reaction parameters, the coverage density of Ag:ZnIn_2_S_4_ nanosheets on the surface of g-C_3_N_4_ nanosheets could be easily controlled.

In-depth investigations of the elemental composition and surface chemical states of the g-C_3_N_4_, Ag:ZnIn_2_S_4_ and g-C_3_N_4_/Ag:ZnIn_2_S_4_ samples, using X-ray photoelectron spectroscopy (XPS), were also performed. Survey spectra of both g-C_3_N_4_ and the g-C_3_N_4_/Ag:ZnIn_2_S_4_ nanocomposites showed peaks for the C and N elements (Fig. S2, ESI†). Additional peaks for Zn, In, and S were also present in the survey spectrum of Ag:ZnIn_2_S_4_ and g-C_3_N_4_/Ag:ZnIn_2_S_4_ nanocomposites (Fig. S2, ESI†). Important data were collected (Fig. 2B–F) from the high-resolution C 1s, N 1s, Zn 2p, In 3d, S 2p XPS spectra of g-C_3_N_4_, Ag:ZnIn_2_S_4_ and g-C_3_N_4_/Ag:ZnIn_2_S_4_ nanocomposites. Thus, in the high-resolution C 1s XPS spectrum of g-C_3_N_4_, two distinctive peaks appeared at 284.6 and 288.2 eV (Fig. 2B), corresponding respectively to accidental hydrocarbons or the sp^2^ graphitic C in C=C bonds and sp^2^ C in N–C=N bonds.^32,33^ Deconvolution of the N 1s XPS spectrum (Fig. 2C) showed three peaks centered at 398.4, 399.1 and 400.5 eV, assignable to the sp^2^ N in N=C–N, tertiary N groups (N–C), and amino functions (C–N–H or C–NH_2_), respectively.^34,35^ The two peaks with binding energies of 1023.0 eV and 1046.1 eV in Fig. 2D correspond to Zn 2p_1/2_ and Zn 2p_3/2_, respectively, indicating the Zn(ii) oxidation state. The high-resolution In 3d spectra (Fig. 2E) show two peaks at approximately 444.7 eV (In 3d_3/2_) and 452.2 eV (In 3d_5/2_), suggesting that the chemical state of the In cation is +3.^36^ In the high-resolution S 2p XPS spectrum of Ag:ZnIn_2_S_4_ (Fig. 2F), the two peaks at binding energies of approximately 161.5 eV and 162.6 eV correspond to the S 2p_3/2_ and S 2p_1/2_ orbitals of S^2−^ ions, which is consistent with ZnIn_2_S_4_. Remarkably, after the loading of Ag:ZnIn_2_S_4_ onto g-C_3_N_4_, in the S 2p XPS spectrum, an obvious shift (by 0.3 eV) of the peaks toward lower binding energy occurred. This indicates that the electron density of S^2−^ ions from Ag:ZnIn_2_S_4_ increased, most likely due to the strong interfacial interactions and to electron transfer from the C atoms in g-C_3_N_4_. Previous experimental results and theoretical calculations from literature confirmed that the S atom preferentially substitutes for the edge N site of the g-C_3_N_4_ lattice to form a C–S bond and, at the same time, excludes the possibility of N–S bonds formation.^37–39^

**Fig. 3:**
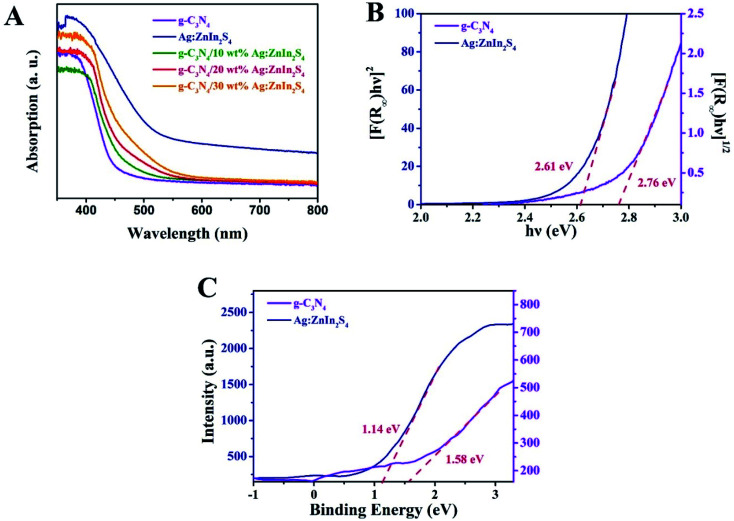
(A) UV-vis diffuse reflectance spectra of g-C_3_N_4_, Ag:ZnIn_2_S_4_ and g-C_3_N_4_/20 wt% Ag:ZnIn_2_S_4_. (B) The bandgap energy and (C) the VB XPS spectra of the g-C_3_N_4_ and Ag:ZnIn_2_S_4_ samples.

The capacity of the as-prepared samples for solar light utilization has been investigated *via* UV-vis diffuse reflectance spectra (DRS). In Fig. 3A, pure g-C_3_N_4_ exhibits an absorption edge at approximately 460 nm, which is consistent with previously reported works.^40^ For Ag:ZnIn_2_S_4_, the absorption edge appears at about 600 nm, indicating strong light-harvesting in both the UV and visible regions. In comparison with pure g-C_3_N_4_, all g-C_3_N_4_/Ag:ZnIn_2_S_4_ nanocomposites displayed absorption edges at longer wavelengths, consonant with the hue of the as-prepared samples that changed from faint yellow to yellowish-brown (Fig. S3, ESI†). Furthermore, based on the UV-vis diffuse reflectance spectra, the bandgap (*E*_g_) energies of g-C_3_N_4_ and Ag:ZnIn_2_S_4_ were extrapolated according to the following equation:*αhν* = *A*(*hν* − *E*_g_)^*n*/2^where *α* is the absorption coefficient, *hν* is the photon energy (eV), *A* is the adsorption constant and *E*_g_ is the bandgap value. As shown in Fig. 3B, the *E*_g_ of g-C_3_N_4_ and Ag:ZnIn_2_S_4_ were extrapolated to about 2.76 and 2.61 eV, respectively. Furthermore, the valence band (VB) positions were acquired by analyzing the VB XPS spectra; thus, the VB of g-C_3_N_4_ and Ag:ZnIn_2_S_4_ were about 1.58 and 1.14 eV, respectively (Fig. 3C). Also, using the formula *E*_g_ = *E*_VB_ − *E*_CB_, the conduction band (CB) potentials of g-C_3_N_4_ and Ag:ZnIn_2_S_4_ were calculated to be −1.18 and −1.47 eV, respectively.

**Fig. 4:**
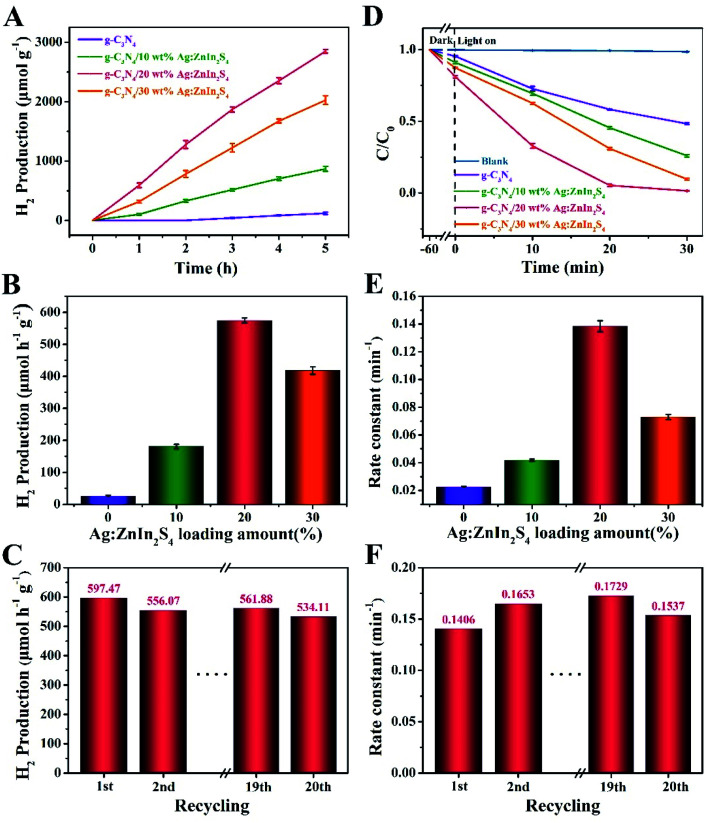
(A) The photocatalytic H_2_ evolution of the g-C_3_N_4_ and g-C_3_N_4_/Ag:ZnIn_2_S_4_ samples for 5 h under visible light irradiation. (B) The photocatalytic H_2_ evolution rates of the above samples. (C) The photocatalytic H_2_ evolution rate, under visible light irradiation over the 5 h reaction time, for twenty successive cycles, using the g-C_3_N_4_/20 wt% Ag:ZnIn_2_S_4_ sample. (D) The photocatalytic degradation of MO over the g-C_3_N_4_, and g-C_3_N_4_/Ag:ZnIn_2_S_4_ samples during 30 min of visible light irradiation. (E) Rates of MO photocatalytic degradation over the above samples. (F) Rates of MO photocatalytic degradation using the g-C_3_N_4_/20 wt% Ag:ZnIn_2_S_4_ sample over twenty successive cycles of 30 min irradiation.

The photocatalytic performance of the as-prepared samples was evaluated by the H_2_ production through water splitting under visible-light irradiation and in the presence of TEOA as a hole scavenger. Fig. 4A shows that a remarkable improvement in the H_2_ evolution was achieved with our nanocomposite photocatalysts. While pure g-C_3_N_4_ displayed a negligible photocatalytic propensity for H_2_ evolution, most likely because of the high recombination rate of photogenerated electrons and holes, all g-C_3_N_4_/Ag:ZnIn_2_S_4_ nanocomposites demonstrated substantially augmented rates of H_2_ evolution. After growing Ag:ZnIn_2_S_4_ nanosheets on the g-C_3_N_4_ nanosheets, the H_2_ evolution rate gradually increased with the Ag:ZnIn_2_S_4_ content. In particular the g-C_3_N_4_/20.0 wt% Ag:ZnIn_2_S_4_ sample exhibited the highest H_2_ evolution rate, of 597.47 μmol h^−1^ g^−1^, which is about 30.73 times higher than that of the g-C_3_N_4_ alone (Fig. 4B). However, a diminished H_2_ evolution rate could be observed when the Ag:ZnIn_2_S_4_ content in the nanocomposites was further increased. The considerably enhanced H_2_ evolution activity of g-C_3_N_4_/Ag:ZnIn_2_S_4_ nanocomposites is consistent with the intimate face-to-face contact between g-C_3_N_4_ and Ag:ZnIn_2_S_4_, which is extremely beneficial for the photogenerated charge transfer. The photostability of the optimal g-C_3_N_4_/20.0 wt% Ag:ZnIn_2_S_4_ nanocomposite was evaluated through photocatalytic H_2_ evolution during prolonged visible-light irradiation (Fig. 4C); no obvious decay was observed during 20 cycles with a total test duration of 100 h of visible-light illumination. Noteworthily, the XRD pattern of the recycled photocatalyst (g-C_3_N_4_/20.0 wt% Ag:ZnIn_2_S_4_) was consistent with that of the initial sample, with no obvious shifts in the positions of the characteristic peaks (Fig. S4, ESI†). Moreover, the TEM image of the recycled photocatalyst (g-C_3_N_4_/20.0 wt% Ag:ZnIn_2_S_4_) proved that the original morphology was maintained (Fig. S5, ESI†). All these results suggest the very good stability of the sample during water splitting.

We also evaluated the photodegradation ability of our as-prepared samples under visible-light irradiation. Methyl orange (MO), an anionic azo dye largely employed in the textile industry and as a pH indicator, was used as a substrate. Along with other dyestuff residues, MO is encountered in industrial effluents and its removal by adsorption^41,42^ or photodegradation^43^ is a persistent problem for wastewater remediation. In photodegradation experiments, each photocatalyst was, in turn, stirred in the MO solution for 1 h under dark conditions to reach the adsorption–desorption equilibrium between the photocatalyst and MO. Under long-term visible-light irradiation, but without the addition of a photocatalyst, the concentration of MO remained constant, suggesting that the photocatalyst plays an essential role in the photocatalytic degradation of MO. Fig. 4D displays the photocatalytic degradation plots, as a function of the light irradiation time, for g-C_3_N_4_ and g-C_3_N_4_/Ag:ZnIn_2_S_4_ nanocomposites. The results prove that the mass percentage of Ag:ZnIn_2_S_4_ in the g-C_3_N_4_/Ag:ZnIn_2_S_4_ nanocomposites greatly affects the photocatalytic degradation performance. With g-C_3_N_4_, only 47.7% of MO can be degraded in 30 min. However, the photocatalytic activity was substantially enhanced when Ag:ZnIn_2_S_4_ was loaded onto the surface of the g-C_3_N_4_. For the optimal g-C_3_N_4_/20.0 wt% Ag:ZnIn_2_S_4_ sample, the removal efficiency of MO increased to 98.5% after 30 min of visible light irradiation. Moreover, the corresponding reaction kinetics was calculated for the degradation of MO (Fig. 4E). Through fitting the data with a first-order reaction model, the apparent reaction rate constant (*k*_app_) was calculated and further normalized with the mass (10 mg) of the catalyst added in the active solution. The highest normalized *k*_app_(*k*) value obtained for g-C_3_N_4_/20.0 wt% Ag:ZnIn_2_S_4_ (0.1406 min^−1^) was about 5.42 times higher than that for the g-C_3_N_4_ nanosheets (0.0259 min^−1^). However, once the surface of g-C_3_N_4_ was coated with excessive Ag:ZnIn_2_S_4_ nanosheets, this synergistic effect was hindered, so the photocatalytic performance of the nanocomposites decreased. We also conducted recycling tests for the photocatalytic degradation of MO to observe the stability and reusability of the g-C_3_N_4_/Ag:ZnIn_2_S_4_ nanocomposites. This experiment was repeated for 20 runs while our optimal sample (g-C_3_N_4_/20.0 wt% Ag:ZnIn_2_S_4_) retained its high activity in MO removal (Fig. 4F), indicating that the as-prepared nanocomposite photocatalysts possess good stability in photocatalysis. Noteworthily, no metal-ion release was detected by ICP-OES in the solution after photocatalysis. To further investigate the stability of the as-prepared nanocomposite, results from XRD and TEM were acquired (Fig. S6 and S7, ESI†), which illustrated no clear changes. These data are further strong evidence for the structural robustness of the as-prepared nanocomposite. The above photocatalytic results indicate that the g-C_3_N_4_/Ag:ZnIn_2_S_4_ nanocomposites are capable of an effective photocatalytic H_2_ evolution and degradation of MO, while also exhibiting good stability under visible light irradiation.

**Fig. 5:**
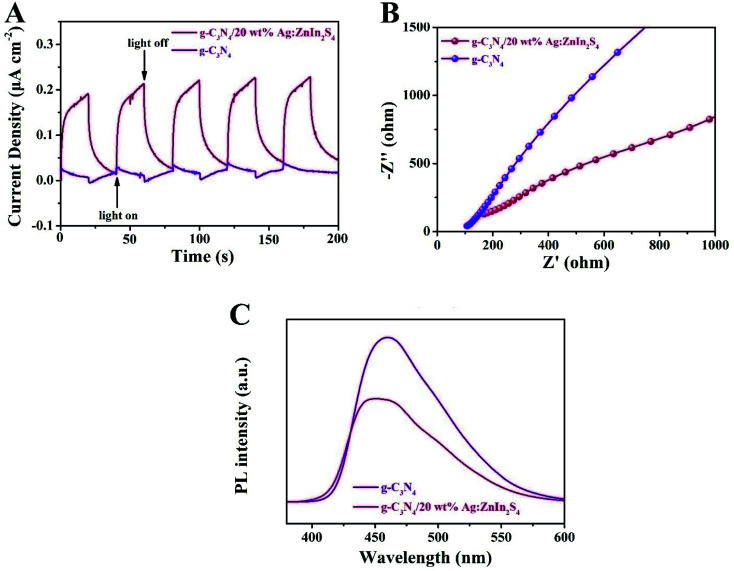
(A) EIS Nyquist plots and (B) transient photocurrent density responses of g-C_3_N_4_ and g-C_3_N_4_/20 wt% Ag:ZnIn_2_S_4_ under visible light irradiation. (C) Steady-state PL spectra of g-C_3_N_4_ and g-C_3_N_4_/20 wt% Ag:ZnIn_2_S_4_.

To ascertain the separation–recombination efficiency of the photogenerated charge of the g-C_3_N_4_/Ag:ZnIn_2_S_4_ nanocomposites, photo/electrochemical characterization was performed as well. As illustrated in Fig. 5A, the g-C_3_N_4_/20 wt% Ag:ZnIn_2_S_4_ nanocomposite exhibited reproducible photocurrent responses for each illumination period with the photocurrent dropping rapidly in the dark during five on–off cycles of light irradiation; better photocurrent responses were displayed as compared to the g-C_3_N_4_. The photocurrent density of g-C_3_N_4_/20.0 wt% Ag:ZnIn_2_S_4_ (0.22 μA cm^−2^) was ∼7.3 times higher than that of g-C_3_N_4_ (0.03 μA cm^−2^), which reveals that the introduction of Ag:ZnIn_2_S_4_ could provide excellent separation of the photoinduced charge carriers. This argument was also confirmed by the electrochemical impedance spectroscopy (EIS) measurements (Fig. 5B), which showed that g-C_3_N_4_/20.0 wt% Ag:ZnIn_2_S_4_ had a smaller arc radius than g-C_3_N_4_. Furthermore, the steady-state PL spectra (Fig. 5C) demonstrated that the g-C_3_N_4_/20.0 wt% Ag:ZnIn_2_S_4_ nanocomposite had a lower PL intensity than g-C_3_N_4_, which revealed that the recombination of electrons and holes was effectively suppressed in the g-C_3_N_4_/20.0 wt% Ag:ZnIn_2_S_4_ sample. In summary, these photo/electrochemical results suggest that the construction of the g-C_3_N_4_/Ag:ZnIn_2_S_4_ nanocomposites can accelerate the separation and migration efficiency of photogenerated e^−^ and h, which are highly favorable for photocatalytic H_2_ evolution and the photocatalytic degradation of MO.

**Fig. 6:**
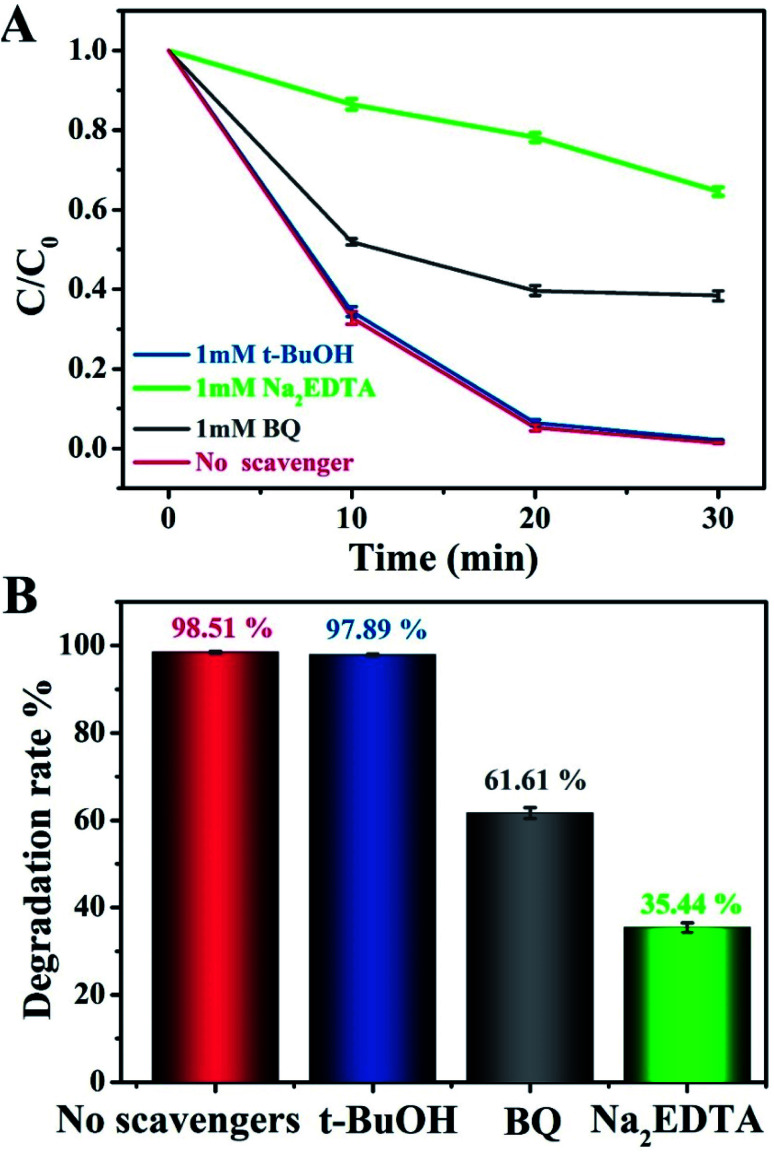
(A and B) Photocatalytic degradation of MO over g-C_3_N_4_/20 wt% Ag:ZnIn_2_S_4_ in the presence of three different scavengers under visible light irradiation.

To evaluate the role of radical species and acquire an in-depth understanding of the photocatalytic reaction mechanism, we conducted control experiments on radical trapping using three different scavengers (Fig. 6). Specifically, BQ, Na_2_EDTA, and *t*-BuOH acted as scavengers for capturing ˙O_2_^−^, h^+^, and ˙OH radicals, respectively.^44–46^ It could thus be seen that the photodegradation activity for MO in the Na_2_EDTA-added system was reduced by 35.3%, as compared to the scavenger-free system, which means that h^+^ played an important role in the reaction. Besides, when BQ was added, the corresponding reaction performance decreased a little, indicating that ˙O_2_^−^ also participated in the process. However, the addition of *t*-BuOH had almost no impact on the photocatalytic activity. This result suggested that ˙OH does not influence the photodegradation of MO.

**Scheme 2:**
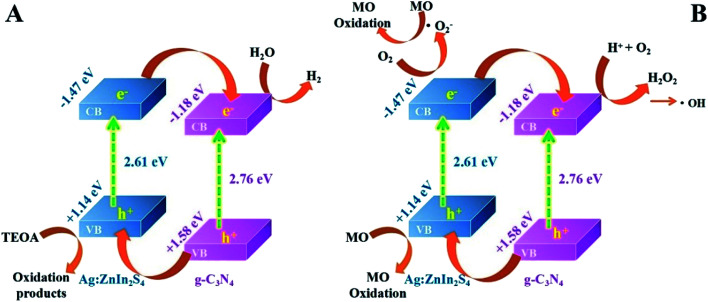
Schematic illustrating (A) the photocatalytic H_2_ production, and (B) the possible mechanisms of the photocatalytic degradation of MO over the g-C_3_N_4_/Ag:ZnIn_2_S_4_ photocatalyst, under visible light irradiation.

Based on the above photocatalytic experimental results, the possible charge-transfer process and the corresponding mechanisms of photocatalytic H_2_ evolution and MO degradation are illustrated in Scheme 2. When irradiated by visible light, both g-C_3_N_4_ and Ag:ZnIn_2_S_4_ could absorb photon energy to generate photoinduced electrons and holes; the photoinduced electrons can move from the VB to the CB state. The CB potential of Ag:ZnIn_2_S_4_ (−1.47 eV *vs.* NHE) has a higher negative value than that of g-C_3_N_4_ (−1.18 eV *vs.* NHE), while the VB potential of the g-C_3_N_4_ (+1.58 eV *vs.* NHE) has a higher positive value than that of the Ag:ZnIn_2_S_4_ (+1.14 eV *vs.* NHE). Considering the positions of the CB and VB of g-C_3_N_4_ and Ag:ZnIn_2_S_4_, the type-II heterostructure could be established. Furthermore, the photoinduced electrons in the CB of Ag:ZnIn_2_S_4_ were transferred rapidly to the CB of the g-C_3_N_4_, while the photogenerated holes in the VB of the g-C_3_N_4_ moved towards the VB of Ag:ZnIn_2_S_4_. Consequently, the photogenerated electron–hole pairs were effectively separated in the g-C_3_N_4_/Ag:ZnIn_2_S_4_ heterostructure and their recombination could be greatly mitigated *via* transfer between g-C_3_N_4_ and Ag:ZnIn_2_S_4_, as confirmed by steady-state PL and PEC measurements. Therefore, the electrons with strong reducibility that accumulated in the CB of g-C_3_N_4_ served as reduction sites to capture H_2_O molecules for H_2_ production (Scheme 2A), while the holes with excellent oxidizability existing in the VB of Ag:ZnIn_2_S_4_ served as oxidation sites to degrade MO (Scheme 2B).

## 4 Conclusions

This paper addresses the environmentally important challenge of creating innovative 2D nanomaterials for high-performance photocatalysis. Covering both fundamental and technological aspects of visible light-enabled hydrogen generation by water splitting and methyl orange degradation, highly active dual nanocomposites were designed, prepared, and tested. In the resulting original 2D/2D heterojunctions, a synergistic enhancement effect of the two building units, g-C_3_N_4_ and Ag:ZnIn_2_S_4_, along with the importance of their weight ratio, is demonstrated. In both application areas, H_2_ generation and MO degradation, the optimal g-C_3_N_4_/20 wt% Ag:ZnIn_2_S_4_ nanocomposite exhibited greatly enhanced photocatalytic activities *vs.* the pure g-C_3_N_4_. The superior photocatalytic performance of g-C_3_N_4_/20 wt% Ag:ZnIn_2_S_4_ was ascribed to an intimate heterojunction interface, an improved band matching of the components and interfacial charge transfer, as well as to the modified electronic structure of ZnIn_2_S_4_ by the Ag^+^ substitutional doping and interstitial doping. Of consequence for the environment and the renewable energy output, the g-C_3_N_4_/Ag:In_2_S_4_ photocatalysts showed excellent stability and durability during recycling. Data acquired in this work on the synthesis and practical use of the so far underexplored materials based on 2D/2D heterojunctions could serve future developments in the area of photocatalysis and specifically, the generation of H_2_*via* visible light irradiation as a cheap alternative energy source. Our results can also contribute to the enhancement of the present, much-needed experience for the convenient removal of harmful substances from contaminated waters through photodegradation.

## Conflicts of interest

The authors declare no competing interest.

## Supplementary Material

RA-010-D0RA06226J-s001
