# Supplementary material for: Designing 2D–2D g-C3N4/Ag:ZnIn2S4 nanocomposites for the high-performance conversion of sunlight energy into hydrogen fuel and the meaningful reduction of pollution
Source: RSC Adv. 2020 Sep 3;10(54):32652–61. doi: 10.1039/d0ra06226j (PMC9056688; doi:10.1039/d0ra06226j)
Supplement: RA-010-D0RA06226J-s001 [file RA-010-D0RA06226J-s001.pdf]

Electronic Supplementary Material (ESI) for RSC Advances.
This journal is © The Royal Society of Chemistry 2020

Electronic Supplementary Material

Designing 2D-2D g-C_3N_4/Ag:$ZnIn_2S_4$ nanocomposites for high-performance conversion of sunlight energy to hydrogen fuel and meaningful reduction of pollution

Yu Gao[#a,b], Kun Qian[#a,b], Baotong Xu[a], Fu Ding[*a,b], Valerian Dragutan[c], Ileana Dragutan[*c], Yaguang Sun[a,b], Zhenhe Xu[*a,b]

[a]The Key Laboratory of Inorganic Molecule-Based Chemistry of Liaoning Province, College of Materials and Engineering, Shenyang University of Chemical Technology, Shenyang 110142, P. R. China.

[b]Key Laboratory of Resource Chemical Technology and Materials (Ministry of Education), Shenyang University Chemical Technology, Shenyang, 110142, PR China

[c]Institute of Organic Chemistry, Romanian Academy, Spl. Independentei 202B, 060023 Bucharest, Romania

E-mail: dingfu@syuct.edu.cn (F. Ding), idragutan@yahoo.com (I. Dragutan), xuzh@syuct.edu.cn (Z. Xu)

[#]These authors contributed equally to this work.

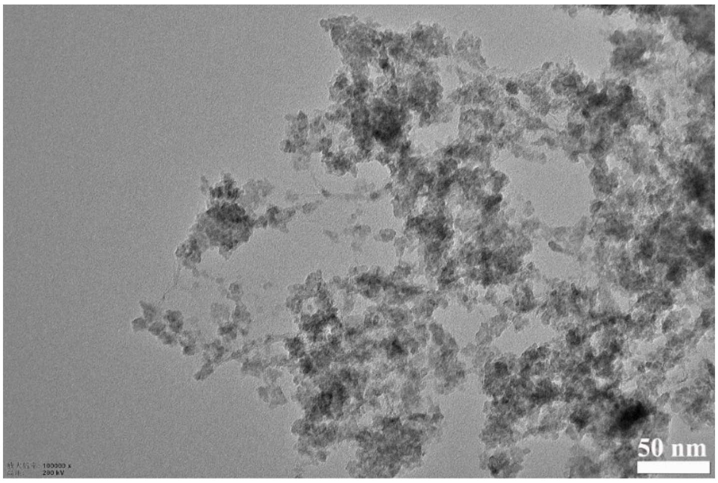

Fig. S1. TEM image of the $Ag:ZnIn_2S_4$ sample.

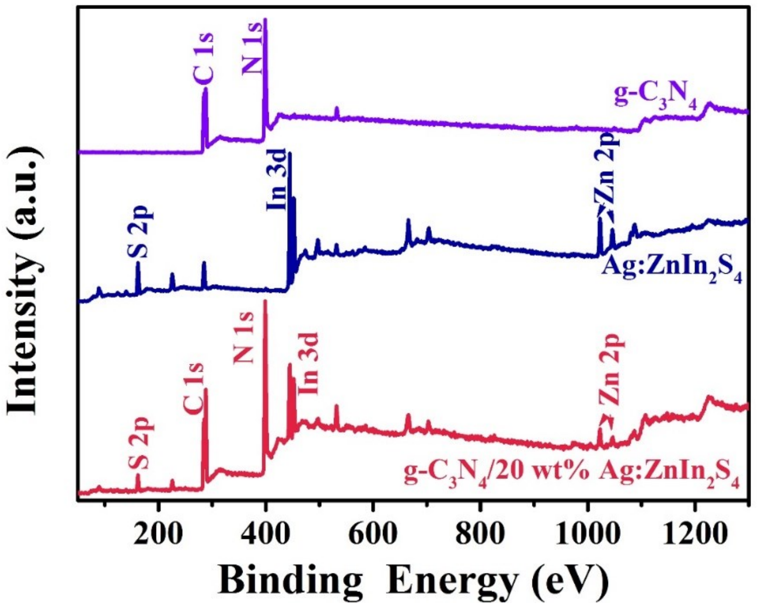

Fig. S2. XPS survey patterns of the g-C_3N_4, Ag:$ZnIn_2S_4$, and g-C_3N_4/20 wt% Ag:$ZnIn_2S_4$ nanocomposites.

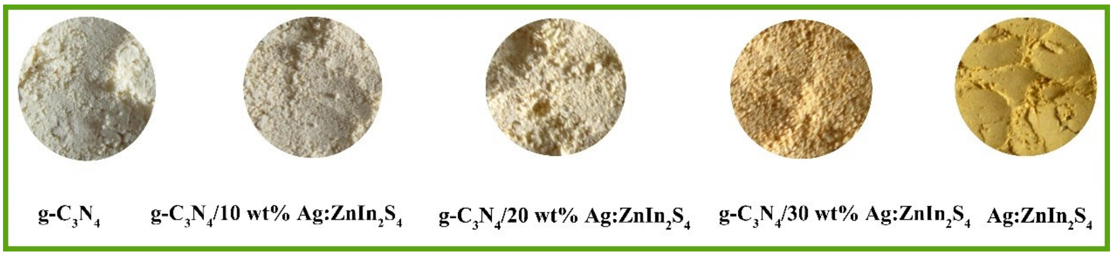

Fig. S3. Photographs of the g-C_3N_4, Ag:$ZnIn_2S_4$, and g-C_3N_4/Ag:$ZnIn_2S_4$ samples.

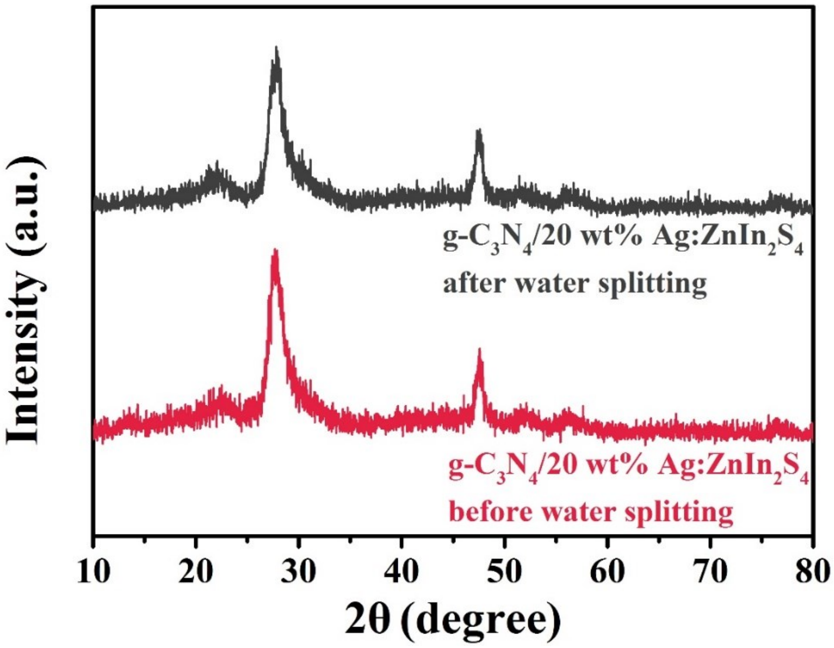

Fig. S4. XRD patterns of the g-C_3N_4/20 wt% Ag:$ZnIn_2S_4$ sample before and after photocatalytic water splitting.

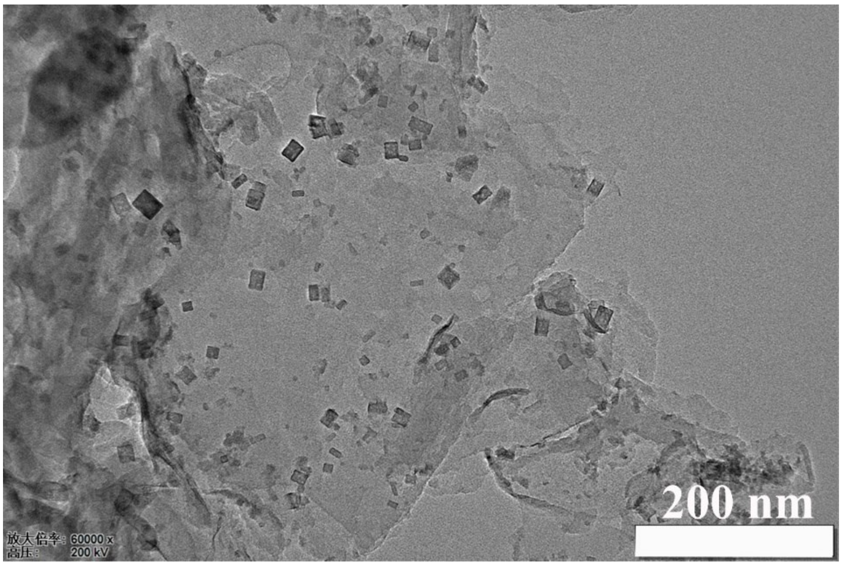

Fig. S5. TEM image of the g-C_3N_4/20 wt% Ag:$ZnIn_2S_4$ sample after photocatalytic water splitting.

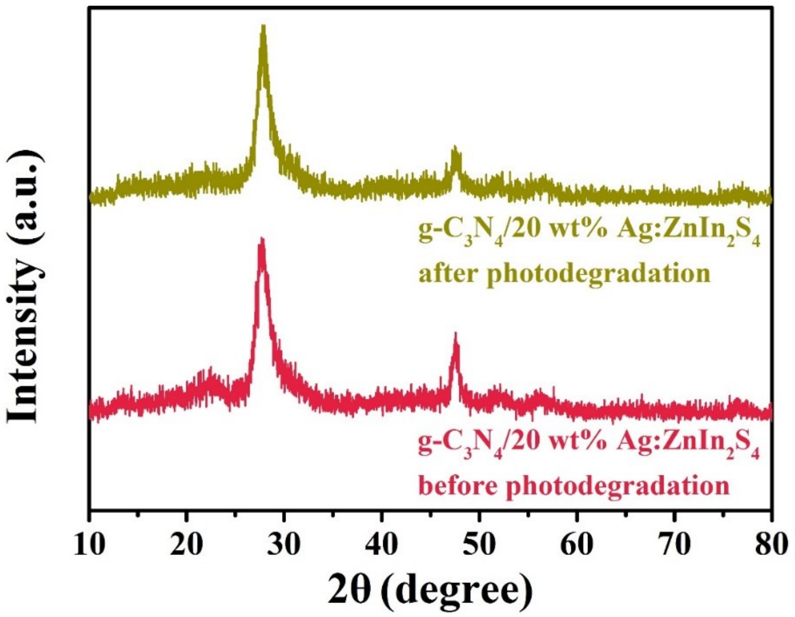

Fig. S6. XRD patterns of the g-C_3N_4/20 wt% Ag:$ZnIn_2S_4$ sample before and after photodegradation of MO.

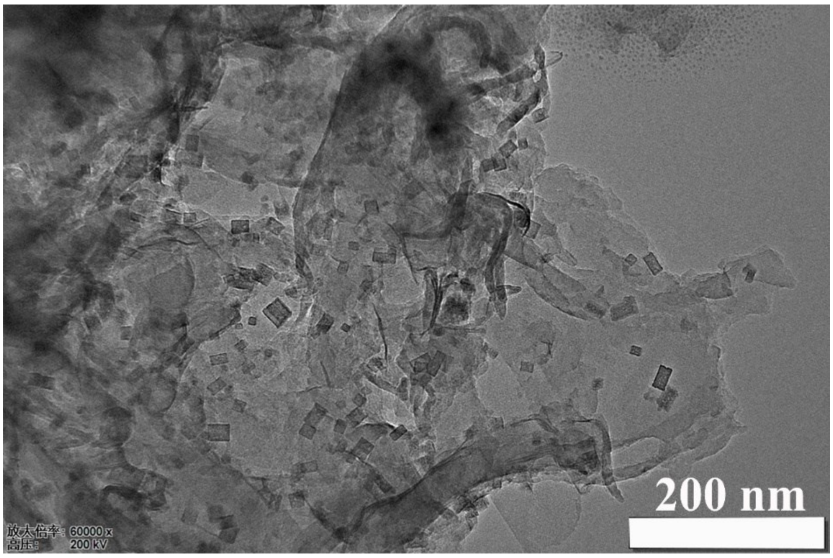

Fig. S7. TEM image of the g-C_3N_4/20 wt% Ag:$ZnIn_2S_4$ sample after photodegradation of MO.
